# Response to Mistry

**DOI:** 10.1038/s41467-020-20175-3

**Published:** 2021-01-12

**Authors:** Jingsong Zhang, Jessica J. Cunningham, Joel S. Brown, Robert A. Gatenby

**Affiliations:** 1 Genitourinary Oncology Department, H. Lee Moffitt Cancer Center, Tampa, FL USA; 2 Integrated Mathematical Oncology Department, H. Lee Moffitt Cancer Center, Tampa, FL USA; 3 Cancer Biology and Evolution Program, H. Lee Moffitt Cancer Center, Tampa, FL USA; 4 Diagnostic Imaging Department, H. Lee Moffitt Cancer Center, Tampa, FL USA

**Keywords:** Cancer screening, Cancer therapy

**Replying to** H. Mistry et al. *Nature Communications* 10.1038/s41467-020-20174-4 (2021)

Mistry (2021) raises three concerns regarding the analysis and reporting^1^ of an adaptive therapy trial using abiraterone on men with metastatic castrate-resistant prostate cancer (mCRPC) (NCT02415621). Briefly, the trial enrolled men presenting with mCRPC who, upon being given abiraterone showed at least a 50% drop in their PSA levels. Upon enrollment abiraterone therapy was withdrawn until the patient’s PSA level returned to its pretreatment level. Abiraterone was then restarted and continued until PSA again dropped to below 50%. During the trial, the patients also underwent CT scans and nuclear medicine bone scans to confirm the PSA response. This on and off cycling of therapy in response to the levels continued until the patient showed PSA and radiographic progression. All men enrolled exhibited at least one complete cycle and the observed outcomes were consistent with those predicted by the evolution-based mathematical model used to design the trial. The patient-specific cycling of abiraterone had two clinical consequences. First, on a per unit time basis, patients received less abiraterone than the continuous standard of care (SOC). Second, the adaptive therapy seemed to prolong progression-free survival and the statistics from the first 11 patients predict superiority but with the obvious need for confirmation by additional clinical investigations.

Mistry (2021) raises legitimate questions regarding our analyses and conclusions that adaptive therapy was superior to SOC. We raised the same questions ourselves. As pointed out, the 16 patients receiving SOC as a separate but contemporaneous cohort, and the 546 patients from a Phase III clinical trial of SOC abiraterone represent imperfect controls. Our trial is a “proof of concept” Pilot trial and, therefore, did not include a randomized control arm. When it appeared highly likely that the men on adaptive therapy were benefitting both from reduced drug use and prolonged progression-free survival, we felt it was important to report our initial results and to provide, as best we could, some statistical support.

The Phase III trial^2^ results provided the largest comparison group, though data did not allow us to identify a subgroup that matched the characteristics of our enrolled cohort. Thus, our trial group’s increased progression-free survival could represent a benefit of adaptive therapy or selection bias for a favorable subpopulation compared to the Phase III trial cohort. For this reason, we included an additional reference group—the contemporaneous cohort of patients who received abiraterone as a frontline therapy for mCRPC and responded with >50% decline of their PSA. All patients included in the contemporaneous cohort were treated at Moffitt and met the eligibility criteria for our adaptive therapy trial but elected SOC treatment and, thus, were not enrolled in the trial. No other selection criteria were applied to this cohort.

Stratifying men with metastatic prostate cancer with respect to prognosis remains the subject of debate. Most investigators would agree that locations of metastasis are prognostic^3^. Gleason’s score may be relevant as well. The locations of metastases and Gleason Scores are well matched between both groups of patients (Tables 1 and 2). The Control and Adaptive therapy groups have very similar Gleason scores (Kruskal–Wallis test statistic of 0.088 yielding *p* = 0.767 based on a Chi-Square distribution, d*f* = 1), and initial pre-abiraterone PSA levels (Kruskal–Wallis test statistic of 0.157 yielding *p* = 0.692 based on a chi-square distribution, d*f* = 1). Liver metastases, which confer a poor prognosis, were excluded in the phase III trial with abiraterone and were similarly excluded in our trial and in the contemporaneous cohort. For mCRPC patients who have been through at least one line of therapy, Hgb and LDH may have some prognostics values. Hgb was >10 for our patients in both cohorts. LDH was not routinely checked in the contemporaneous cohort. Realizing that this still is not a randomized control, we were conservative in our analysis by assuming that those men receiving adaptive therapy who had not yet progressed did so at the time we were writing the preliminary report^1^ (only 2 of 11 actually had). AT was shown to be superior and we estimated that the median time to progression would not be lower than 27 months.

**Table 1:** Gleason scores and metastatic locations in the adaptive therapy and standard therapy cohorts.

	Control	Adaptive
Mean Gleason	7.85	7.94
Range Gleason	[7, 10]	[6, 10]
Mean Pre Abi PSA	36.52	29.7
Range Pre Abi PSA	[2.71, 93.4]	[1.46, 109.4]
*AJCC 7th Ed. M stage*		
M1a	1	1
M1b	14	14
M1c	1	1

**Table 2:** Information on each patient within both cohorts.

Subject ID	Gleason sum	Site of metastasis	Pre Abi PSA (ng/ml)
01	7 (4 + 3)	Bone	83.55
02	Unknown	Bone, lung	6.37
03	7 (4 + 3)	Bone	3.04
04	9 (4 + 5)	Bone	4.48
05	7 (4 + 3)	Bone	54.38
06	9 (4 + 5)	Bone	2.71
07	7 (4 + 3)	Bone	4.04
08	8 (5 + 3)	Bone	23.59
09	Unknown	Bone, lymph node	56.71
10	8 (4 + 4)	Bone, lymph node	19.79
11	7(4 + 3)	Lymph node	6.64
12	7 (3 + 4)	Bone	4.38
13	10 (5 + 5)	Bone	91.73
14	7	Bone, lymph node	78.9
15	Unknown	Bone, lymph node	50.6
16	9 (4 + 5)	Bone, lymph node	93.4

Subject ID	Gleason Sum	M1 sites	Pre Abi PSA (ng/ml)
1001	8 (4 + 4)	Bone, soft tissue	6.06
1002	8 (4 + 4)	Bone	58.57
1003	9 (5 + 4)	Bone, lymph node	68
1004	10 (5 + 5)	Bone	1.64
1005	7 (4 + 3)	Lymph node	95.86
1006	8 (4 + 4)	Bone	15.25
1007	6 (3 + 3)	Bone	109.4
1009	6 (3 + 3)	Bone	13.55
1010	7 (3 + 4)	Bone	17.33
1011	9 (4 + 5)	Lymph node, soft tissue	2.42
1012	8 (4 + 4)	Bone	4.17
1014	7 (4 + 3)	Bone	11.83
1015	8 (4 + 4)	Bone	7.25
1016	7 (3 + 4)	Bone	34.02
1017	9 (4 + 5)	Bone	21.59
1018	Unknown	Bone	36.54
1020	10 (5 + 5)	Bone	1.46

Three years have now passed since the preliminary report. All patients in the contemporaneous cohort have progressed while four remain on the adaptive therapy trial. The median time to progression in the adaptive therapy group remains consistent with the prediction in the preliminary report^1^. A paper outlining fully describing the results is under preparation

Although our original plan was to confirm our findings with a randomized study, the SOC for patients with metastatic prostate cancer has changed with early utilization of abiraterone in the metastatic castrate sensitive setting. We are currently conducting a second adaptive therapy trial (NCT03511196) in metastatic castration sensitive prostate cancer (mCSPC) utilizing the same evolutionary game theory model reported in ref. ^1^.

The impacts of our original adaptive abiraterone study seem to be fourfold. First, it has significantly extended progression-free survival in a small cohort of men. Some of these men, as they have progressed, remained progression-free sufficiently long to move into new clinical trials that offer them novel opportunities. Second, the trial has inspired deeper investigations into the mathematical models of adaptive therapy with a greater understanding of the clinical circumstances in which this strategy is likely or unlikely to be effective. Third, it has promoted lockstep between concepts of evolutionary ecology, mathematical modeling, and novel clinical application. An important part of this integration, now underway for our trial cohort, is that the mathematical model allows a detailed investigation of the clinical course of each patient in addition to traditional cohort analysis. Fourth, by focusing attention on the evolutionary dynamics of cancer treatment and the feasibility of perturbing those dynamics using mathematical models and computer simulation to improve outcomes, an evolutionary tumor board (ETB) has been established at Moffitt. Chaired by two clinical oncologists, the ETB integrates faculty mathematicians and evolutionary biologists into the traditional clinical membership of multidisciplinary cancer conferences to provide evolution-based simulations for individual patients being discussed. The ETB will then compare model predictions to the patient’s subsequent clinical course as an “*n* of 1” trial (NCT04343365).

## Data Availability

Data is available upon request.
